# Scanning Behavior in Echolocating Common Pipistrelle Bats (*Pipistrellus pipistrellus*)

**DOI:** 10.1371/journal.pone.0060752

**Published:** 2013-04-08

**Authors:** Anna-Maria Seibert, Jens C. Koblitz, Annette Denzinger, Hans-Ulrich Schnitzler

**Affiliations:** Institute for Neurobiology, University of Tübingen, Tübingen, Germany; University of Western Ontario, Canada

## Abstract

Echolocating bats construct an auditory world sequentially by analyzing successive pulse-echo pairs. Many other mammals rely upon a visual world, acquired by sequential foveal fixations connected by visual gaze saccades. We investigated the scanning behavior of bats and compared it to visual scanning. We assumed that each pulse-echo pair evaluation corresponds to a foveal fixation and that sonar beam movements between pulses can be seen as acoustic gaze saccades. We used a two-dimensional 16 microphone array to determine the sonar beam direction of succeeding pulses and to characterize the three dimensional scanning behavior in the common pipistrelle bat (*Pipistrellus pipistrellus)* flying in the field. We also used variations of signal amplitude of single microphone recordings as indicator for scanning behavior in open space. We analyzed 33 flight sequences containing more than 700 echolocation calls to determine bat positions, source levels, and beam aiming. When searching for prey and orienting in space, bats moved their sonar beam in all directions, often alternately back and forth. They also produced sequences with irregular or no scanning movements. When approaching the array, the scanning movements were much smaller and the beam was moved over the array in small steps. Differences in the scanning pattern at various recording sites indicated that the scanning behavior depended on the echolocation task that was being performed. The scanning angles varied over a wide range and were often larger than the maximum angle measurable by our array. We found that echolocating bats use a “saccade and fixate” strategy similar to vision. Through the use of scanning movements, bats are capable of finding and exploring targets in a wide search cone centered along flight direction.

## Introduction

Bats use echolocation for spatial orientation and foraging. Their echolocation signals are emitted through either the mouth or nostrils, and form a directional sonar beam [1], [2]. The aiming of this sonar beam is determined by the head position during sound emission. The directional beam covers only a partial field of the world around a bat. It has been estimated that bats can react to prey flying within a “search cone” which is up to 120–150° wide [3], [4]. However, it is not known how head and beam are oriented when bats perform specific echolocation tasks in the field. Do bats always direct head and beam in flight direction so that the directionality of the beam alone determines the search cone or do they probe the environment with scanning movements, thereby increasing search volume?

To address these questions it is necessary to determine the sonar beam aiming in free- flying bats. This is only possible if the flight path of a bat is known and their signals are sampled with sufficient spatial resolution such that the reconstruction of the beam is possible and that its aiming direction relative to the bat’s flight path can be determined.

The possibility of bats actively changing their sonar beam aiming has been derived from single microphone recordings in the field. Successive signals often show distinct changes in signal amplitude. These could be indicators for scanning movements [4], [5], [6], [7]. However, this periodic variation in bats has been argued to be due to deliberate amplitude variation as a strategy for correct pulse-echo pair assignation [8]. In one of the few studies examining beam-aiming behavior in the field, three stationary microphones were used to conclude that *Eptesicus serotinus* point their beam downward when flying high and forward when flying at lower altitudes [9].

In the laboratory, scanning studies have been made with linear microphone arrays where trained bats directly approached prey [10], [11], [12], [13] or after flying through a gap [14]. The array used in these experiments consisted of microphones arranged on a plane, in a U-shaped arrangement along three walls of a flight room. The reconstructed beams were therefore only horizontal cross sections of the beam, thus the observed scanning movements described only the horizontal movement of an apparent beam maximum. Vertical movements were not indicated. Nevertheless, the experiments provided interesting results on the scanning behavior of bats. When bats were trained to fly through a gap *Eptesicus fuscus* displayed sequential scanning of the two edges of the opening [14]. When bats searched for prey in the laboratory, scanning movements in the horizontal plane were also described but not quantified [10]. During the approach to prey *E. fuscus* directed the apparent beam maximum on the prey. This led to the general conclusion that bats lock their real beam maximum onto a single target of interest while approaching it. More recently, a two-dimensional 16 microphone array was used to determine the sonar beam aiming in landing. *E. fuscus.* The bats attended to the target of interest by keeping the sonar beam locked onto the landing site during the approach beginning at distances of 1–2 m [15].

From analyses from head aiming in photographs or videos, it was also concluded that bats direct their sonar beam towards a target when approaching it [16], [17] or follow moving targets with the beam while sitting on a platform [18].

Sequential acquisition of sensory information by the scanning of an environment with a sensory organ has been studied mainly in the visual systems of humans and animals. Humans look at or search for objects by integrating head and eye movements to visual gaze saccades [19]. After each saccade they fixate a new area of interest and retain the visual image on the fovea. Such foveal fixations take 200–300 ms on average [20]. The gaze saccades that connect such fixations are task-specific, and depend on the observer’s behavioral goals [21].

Ghose and Moss (2003) made an analogy to visual gaze by terming the bats’ aiming with their sonar beam as an “acoustic gaze”. Surlykke et al. (2009) compared the scanning behavior in bats to active visual scanning. We also hypothesize that echolocating bats use a sequential “fixate and saccade” strategy comparable to visual systems [22]. To acquire information on the acoustic world around them, bats are able to move their acoustic gaze from one pulse or fixation to the next. Bats continuously emit echolocation signals and the analysis of each pulse-echo train delivers information comparable to the visual information delivered by each foveal fixation. By shortening the interval between pulses, bats can increase the rate of fixations and with it the update rate of new information.

In this work we were interested in the scanning behavior of pipistrelle bats (*Pipistrellus pipistrellus*) flying in the field in open and edge space. Our hypothesis is that bats in the field make scanning movements, or “acoustic gaze saccades”, which are comparable to visual scanning. We assume that their scanning behavior is also task specific. We therefore determined the scanning behavior of bats in relation to performing various tasks including searching for prey, orienting in space, and approaching an obstacle at three different recording sites (referred to as forest, farm, and garden).

## Results

### Flight and Echolocation Behavior

**Figure 1:**
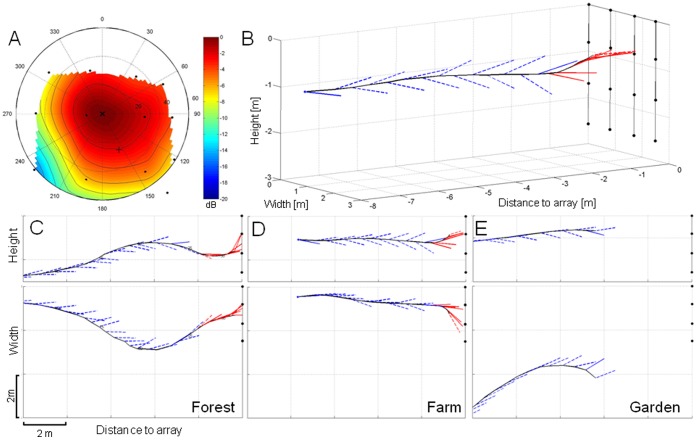
Beam reconstruction and exemplary flight paths towards the array at different edge habitat recording sites. Reconstructed sonar beam (A) and three-dimensional display of a flight towards the microphone array (B). The SPL in (A) is color-coded and indicates the beam form relative to the beam maximum in the center of a polar plot. The black dots mark the positions of the microphones, the cross representing the flight direction. C–E depict side views (upper row) and overhead views (lower row) of exemplary flights towards the microphone array which was positioned ∼ 1.6 m above ground at the three recording sites: (C) forest road, (D) farm, (E) garden. The flight paths are depicted as black lines. Each of the blue (search calls) and red (approach calls) vectors begins at the bat’s position at the time of call emission and points towards the calculated position of the reconstructed apparent (dotted line) or real (solid line) beam maximum on the array plane. The black dots represent the 16 microphones of the array. **Note:** the apparent vectors do not indicate the real aiming of the beam, and the angle between successive vectors not the real scanning angle. The real scanning angles may be much larger than the apparent scanning angles on this graph. The real scanning angle is only indicated if the beam maxima were within the array.

**Figure 2:**
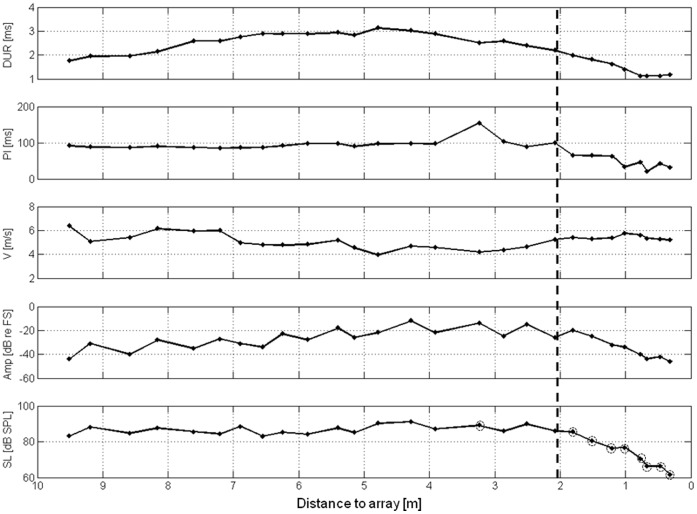
Echolocation behavior and flight speed of a *P. pipistrellus* flying towards the microphone array. The parameters pulse duration (DUR), pulse interval (PI), flight speed (V), max. signal SPL measured at the upper right microphone of the array (amplitude in dB relative to full scale), and source level (SL in dB SPL *re* 20 µPa at 1 m) of one typical flight plotted over distance to the array. In the lowest graph, real source levels (SL) of signals within the array are marked by circled points, all other values indicate apparent source levels (ASL). The dashed line indicates the beginning of the approach.

In edge space, the reconstructed flight paths of *P. pipistrellus* flying towards the microphone array and the corresponding sound sequences allowed for a discrimination of two distinct behaviors at all three recording sites. Beyond a ∼2 m distance to the array, bats exhibited flight directly towards the microphone array with roughly constant flight speed (Figure 1B–E). Search calls with interspersed hunting sequences indicated that the bats were foraging. From these results, we concluded that the bats were in search flight and that their behavior corresponded to search behavior. At ∼2 m in front of the array, the bats switched from search to approach behavior. During approach, bats made avoiding maneuvers resulting in passes around or through the array (Figure 1B–E). Signal parameters during approach were characterized by higher bandwidth, shorter duration, shorter pulse intervals, lower terminal frequency, higher pulse density, and reduction in source level (SL) (Figure 2). In open space, bats were foraging at heights several meters above ground level. As these recordings were obtained with a single microphone we could not determine exact locations and flight paths of individual bats.

#### Search flight in edge and open space

In edge space, the bats’ behavior during search flight differed between the three recording sites. In the forest, individuals flew nearly straight at heights between 0.5–4.5 m and directly towards the microphone array (Figure 1C). Average flight speed was 5.4 m/s. Mean call duration was 3.1±0.7 ms at a mean pulse interval of 91.2±7.5 ms. Terminal frequency of calls was 48.0±1.3 kHz, and the source level was 84.5±2.1 dB SPL *re* 20 µPa at 1 m (Table 1). On the farm, individuals flew around a corner and passed along a house wall at a close distance and at heights between 1–3 m above ground (Figure 1D). Bats were also observed circling, presumably in search of prey. Mean call duration was 3.0±0.6 ms with a pulse interval of 89.6±4.3 ms. Terminal frequency was 46.5±3.4 kHz, and the source level was 93.8±5.0 dB (Table 1). In the garden, the bats were usually foraging, and would circle at heights of between 1–3 m above ground in front of the microphones (Figure 1E). Mean call duration was 3.8±1.0 ms at a mean pulse interval of 86.4±3.3 ms. Terminal frequency was 48.4±1.3 kHz, and the source level was 95.8±2.1 dB SPL (Table 1). Different recording localities compared to each other showed a significant difference in mean SPL with the forest site with 85 dB having the lowest SPL of all three recording sites (Table 1., F_2,29_ = 46.00, *p*<0.0001; Tukey HSD, *p*≤0.05).

**Table 1:** Parameters of pipistrelle bat search signals.

		Forest	Farm	Garden	Open Space
		*(n = 183, N = 15)*	*(n = 82, N = 8)*	*(n = 137, N = 9)*	*(n = 225, N = 22)*
**Duration [ms]**	**mean** (±SD)	**3.1** (±0.7)	**3.0** (±0.6)	**3.8** (±1.0)	**6.2** (±0.8)
	min	2,3	2,1	2,0	4,0
	max	4,5	3,8	4,7	7,5
		*(n = 139, N = 15)*	*(n = 67, N = 8)*	*(n = 107, N = 9)*	*(n = 102, N = 21)*
**Pulse interval [ms]**	**mean** (±SD)	**91.2** (±7.5)	**89.6** (±4.3)	**86.4** (±3.3)	**97.7** (±4.7)
	min	80,6	85,2	81,6	89,5
	max	102,6	97,3	92,6	104,6
		*(n = 183, N = 15)*	*(n = 82, N = 8)*	*(n = 137, N = 9)*	*(n = 225, N = 22)*
**Terminal frequency [kHz]**	**mean** (±SD)	**48.0** (±1.3)	**46.5** (±3.4)	**48.4** (±1.3)	**45.3** (±1.6)
	min	45,5	38,7	47,0	43,2
	max	50,2	49,0	51,5	49,1
		*(n = 183, N = 15)*	*(n = 82, N = 8)*	*(n = 137, N = 9)*	
**Source level [dB SPL]**	**mean** (±SD)	**84.5** (±2.1)	**93.8** (±5.0)	**95.8** (±2.1)	N/A
	min	81,0	84,7	90,9	N/A
	max	87,5	98,8	97,9	N/A

Parameters of search signals in *Pipistrellus pipistrellus* emitted when flying towards the array at three edge habitat recording sites and in open space. For each sequence only the mean of the contained parameters were used for statistics to reduce pseudoreplication. In edge space recordings, only calls within a distance of 3–10 m from the array were analyzed, so as to exclude approach signals. *n* = number of calls used to calculate the corresponding mean. *N* = number of recorded sequences.

In open space, where bats do not react to the background in their echolocation behavior [23], a mean signal duration of 6.2±0.8 ms was significantly longer than the duration in the three edge space situations (F_3,50_ = 64.14, *p*<0.0001; Tukey HSD, *p*≤0.05). The pulse interval with 97.7±4.7 ms was also significantly longer than in the other three locations (F_3,49_ = 11.24, *p*<0.0001; Tukey HSD, *p*≤0.05) (Table 1). Recordings with one microphone did not allow for a determination of the SL.

#### Approach flight in edge space

The behavior of bats during approach flights at the three recording sites was similar to that previously described for pipistrelle bats [2]. In the forest, most bats avoided the array and flew around it, while only one flew through the array. At the farm no bats passed through the microphone array. They mostly passed above or through the gap between the array and the house wall. Only a few flew around the array on the right side. During approach the bats usually kept a speed of 4–6 m/s. However, when bats made sharp avoiding maneuvers or passed between the microphones, flight speed was reduced to approx. 2.5 m/s. At the garden site, bats rarely approached the array.

Signal parameters during approach stereotypically changed to a shorter duration, shorter pulse intervals, and a reduction in SL. Call duration and pulse interval were reduced at the closest point in front of the array to ∼ 1 ms and 50 ms, respectively. In halving the distance to the array, the SL was reduced by ∼ 8 dB SPL (*n* = 182; y = 25*log*(distance)+75; R^2^ = 0.45).

### Scanning Behavior in Search Flight

**Figure 3:**
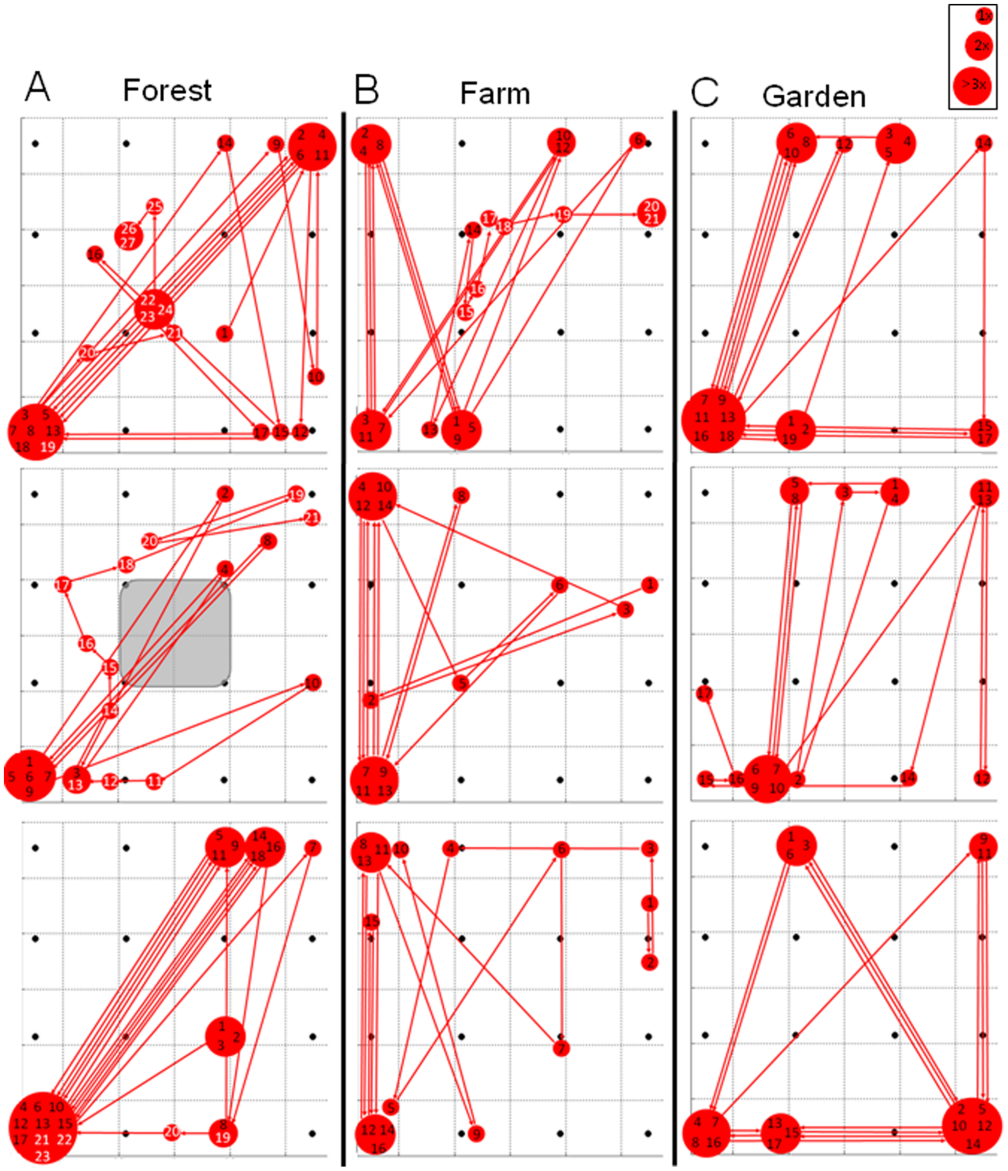
Scan paths at three edge habitat recording sites. Scanning behavior during three typical flights at each of the three edge habitat recording sites. (A) forest road, (B) farm, (C) garden. The scanning behavior is indicated as pulse-to-pulse scan path of the calculated apparent or real beam maximum on the array plane. Successive pulses are identified by either black (search) or white (approach) numbers. The larger the circle around the numbers, the more calls were pointed at this spot. All beam maxima pictured on the outer edges of the array are apparent beam maxima; real maxima are within the array. The grey square at the forest site depicts the location where a single bat flew through the array.

**Figure 4:**
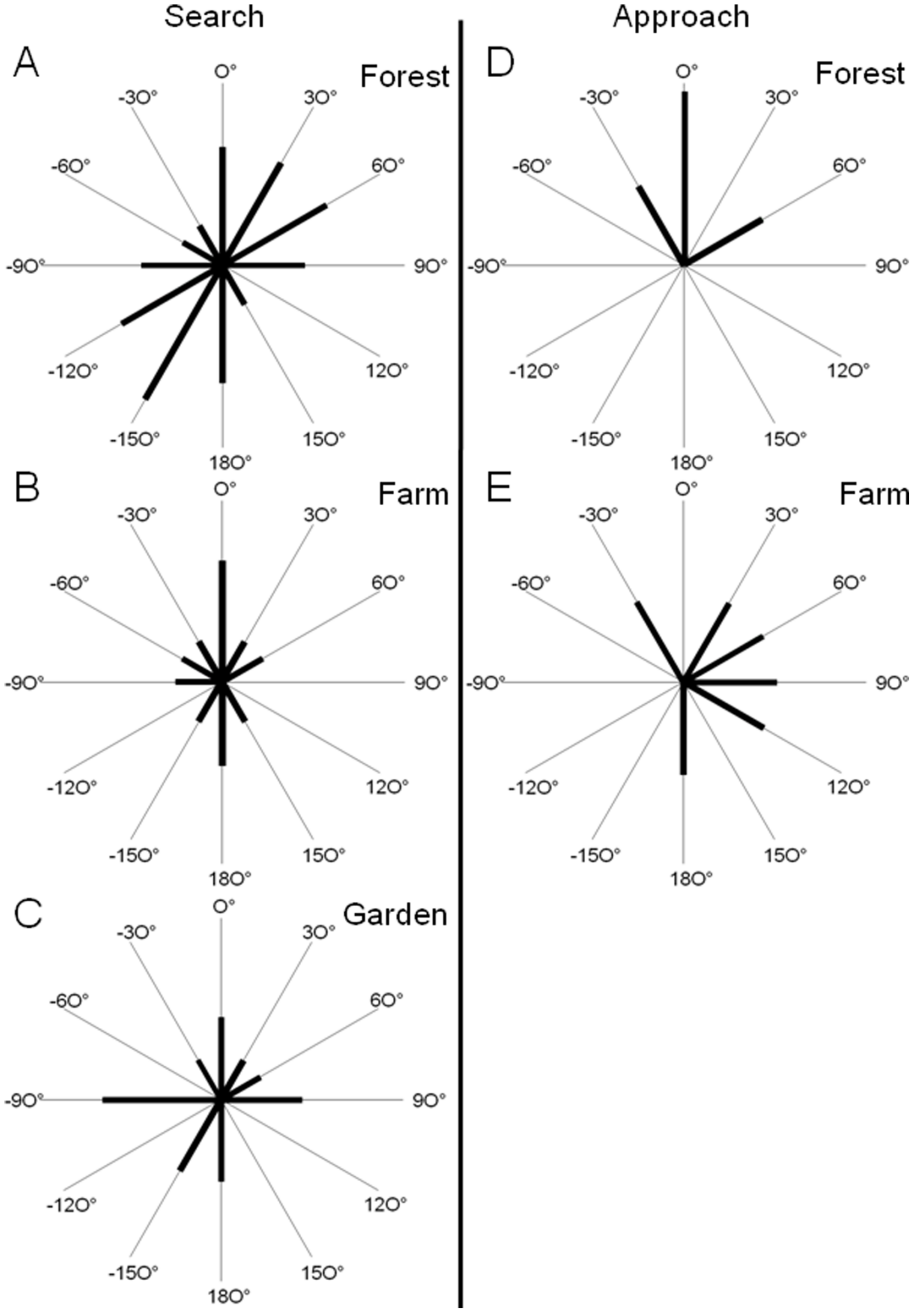
Angular directions of scan paths on the array. Polar histogram of angular directions of apparent and real scan paths from sequences recorded at the three edge habitat recording sites. Each of the indicated directions contains all scan path directions within a ±15° wide sector. The lengths of the black lines indicate numbers of observations. A–C depict scan path directions of search signals, and D and E represent scan path directions of approach signals. (A) forest road (180 search calls during 14 sequences), (B) farm (92 search calls during 8 sequences), (C) garden (78 search calls during 6 sequences), (D) forest road (60 approach calls during 12 sequences), (E) farm (21 approach calls during 4 sequences).

**Figure 5:**
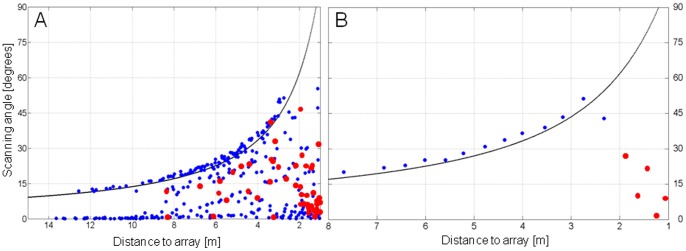
Apparent and real scanning angles. Apparent and real scanning angles of pipistrelle bats flying towards the microphone array. Angles are measured from the bat’s position and between successive apparent or real beam maxima on the array plane. The black line indicates the calculated maximum angle in the vertical plane that can be measured with the microphone array according to its dimensions and distance from it. (A) Scanning angles of 414 calls measured during 18 flight sequences at the forest and the farm. (B) Scanning angles of a single approach sequence at the farm. Apparent (blue dots) and real (red dots) scanning angles are indicated.

Reconstructed flights with aiming vectors (Figure 1) describe the scanning behavior of bats while flying towards the microphone array. Bats often moved their beam back and forth within a search cone pointing in flight direction. The scan paths of the apparent and real beam maxima on the array plane also characterize the scanning behavior (Figure 3). The comparison of the scan paths at the three recording sites revealed distinct site-dependent differences. The differences in the patterns of the angular orientation of the beam movements indicate that the scanning behavior was site- and therefore task-specific (Figure 4A–C). On the forest road, scanning movements were mostly diagonal between the upper right corner of the array and the lower left corner. Scanning movements up and down occurred less often, with movements to the left and right side occurring the least (Figure 4A). At the farm, scanning movements up and down were dominant, followed by diagonal scanning movements. Scans to the sides were rarely performed (Figure 4B). At the garden, scanning movements to the left and right dominated, but movements up, down, and diagonal also occurred (Figure 4C). In search flight, pipistrelle bats often scanned at angles larger than those that could be observed by our array, resulting in apparent scanning angles (Figure 5A). The largest apparent angle measured was 51°, and the largest real angle was 42°. Successive calls sometimes pointed in the same direction, and are therefore assigned a scanning angle of 0°.

**Figure 6:**
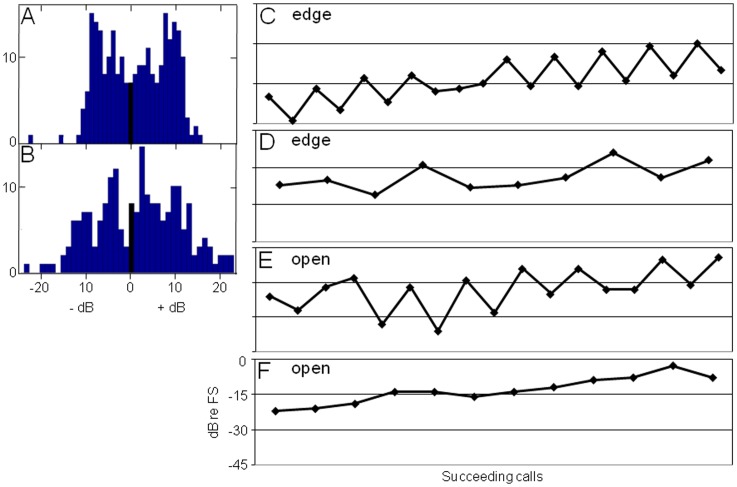
Comparison of scanning behavior in edge and open space. Scanning behavior of bats flying in an edge and open space derived from single microphone recordings. (A) Histogram of differences in SPL between consecutive signals in edge space (249 calls during 15 sequences) as compared to (B) histogram of differences in SPL in open space (203 calls during 20 sequences). Exemplary call sequences with distinct alternating changes of signal SPL in edge space (C) and in open space (E). Examples with more irregular changes in signal SPL for edge space (D) and for open space (F).

The single microphone recordings from open space revealed clear amplitude differences in successive calls in 90% of the recordings, totaling more than 200 calls (18 of 20 sequences, Figure 6B). The mean difference was 8.1±5.3 dB, but differences ranged as high as 24 dB. When examining recordings of a single microphone in the array, we also found clear and often alternating variations of amplitude of successive search signals in 96% of sequences from the three sites in edge space (76 of 79 sequences, Figure 6A). The mean difference of consecutive calls was 6.7±3.7 dB, with a maximum difference of 23 dB. These distinct variations in search flight can be explained by the documented scanning movements, likely due to the fact that a single microphone picked up different parts of the sonar beam of successive pulses. In both situations, we found distinct alternating changes of signal SPL (Figure 6C for edge space and (E) for open space) and less obvious, less regular changes in signal SPL among succeeding calls (Figure 6D for edge space and (F) for open space).

### Scanning Behavior in Approach Flight

During approach the scan path of the real beam maximum remained mainly within the array plane, e.g., the scan path moved around the inner four microphones forming the gap which was passed by an individual (Figure 3A center plot) or the scan path moved towards the right side as an individual passed the array (Figure 3B upper plot).

In the forest, the angular direction of most scan paths pointed upwards (Figure 4D). Here, bats often passed the array by flying over it. At the farm, the scan path angles pointed more to the right side where most of the bats passed (Figure 4E). At the garden, we did not obtain any approach calls as the bats did not pass the array. Closer than ∼ 2 m to the array, most real scanning angles were below 10° (Figure 5A and B), thus the scanning movements during approach were smaller than in search flight.

## Discussion

To our knowledge, this study is the first to describe the scanning behavior of an echolocating bat species (*P. pipistrellus*) when flying under natural conditions in the field. Using a planar 16-microphone array allowed us to not only reconstruct flight paths and assign source levels, but also to reconstruct the real aiming direction of the sonar beam (provided its maximum was within the array). Often the maxima of the recorded signals were not within the array such that it was only possible to reconstruct horizontal or vertical cross sections of the beam along the row of microphones with the apparent beam maximum. The scan path of apparent beam maxima also revealed how bats moved the beam and how these movements were influenced by the echolocation tasks the bats performed.

### Flight and Echolocation Behavior during Different Echolocation Tasks

The echolocation tasks of bats depend on where they search, find, and acquire food. These tasks differ depending on the habitat type and the foraging situation.

Open space is defined as the foraging habitat where bats do not react to background targets [23]. We assume this lack of reaction indicates that spatial orientation in relation to the ground is not an important echolocation task, and that the bats mainly search for insects and increase their chances to detect prey by choosing suitable signals from their signal repertoire. The distinctly longer pulse intervals and signal durations in our single microphone recordings indicate that the bats were foraging in open space. The observed echolocation behavior was similar to that reported for bats foraging in the open [24].

In edge space, bats adjust their echolocation behavior to the background echoes by shortening the pulse duration, increasing the bandwidth, and reducing the SPL of their signals with decreasing distance to the background [1], [4], [17], [25]. When foraging in edge situations bats have to simultaneously perform two different tasks. They perform spatial orientation tasks while navigating along the background targets and reacting to unknown obstacles such as our microphone array. Additionally, they search for flying insects, a task that may be influenced by clutter echoes from the background. The observed echolocation behavior in our study was similar to that reported for bats foraging in edge situations [24]. However, behavior differed between the three recording sites, which may reflect differences in the spatial orientation task, e.g., differences were found in the source levels. In more open edge habitats including the garden and farm sites, bats emitted signals averaging 96.1 and 95.4 dB, respectively. In the more closed gap situation at the forest site, signals only reached an average of 84.8 dB. We assume that the bats lowered their calling amplitudes to reduce background echoes in the more closed situation, whereas in the more open situations at the farm and the garden, the bats use higher SLs. A similar explanation for the lowering of the SLs of *Macrophyllum macrophyllum* was used when flying near vegetation [26]. Site-dependent differences in SL were also found and explained by different foraging situations or individual differences [8].

When approaching the microphone array, bats had to perform an additional spatial orientation task. They had to collect all of the information necessary to guide their flight path around or through the unknown obstacle. The switch to approach behavior found in our study is in accordance with earlier findings in pipistrelle bats that react to prey or targets at a distance of ∼ 2 m [17], [24]. During the approach to the array, flight speed was reduced and signal duration and pulse interval decreased in a stereotypical manner, while SL was reduced by an average of 8 dB per halving of distance. This is slightly higher than the previously reported 6 dB per halving of distance to the target [27], [28]. However, [15] also reported SPL reductions of 9 dB per halving of distance in landing *E. fuscus*.

### Scanning Behavior during Different Echolocation Tasks

We found that pipistrelle bats often scan their environment by changing the sonar beam direction from pulse to pulse, most likely by rapid head movements. The beam can be aimed in all directions within a rather wide cone around the flight direction and it is not only moved in the horizontal plane, as indicated by recordings of the apparent beam maxima with horizontal microphone arrays [10], [11], [13], [14]. The scanning patterns ranged from almost no beam movement to very large scanning movements between successive pulses. The largest real scanning angle we could measure with our array was 42°. Many calls had maxima outside our array, indicating that even larger angles were possible.

Sequential scanning behavior of a sensory system is best understood in vision. The aiming of the sonar beam can be compared to a visual gaze. Therefore we also use the term “acoustic gaze” [10]. The visual gaze system produces head and eye movements, or gaze saccades [19], so that the object of interest can be examined with the fovea. Visual information is sequentially acquired. Once a target of interest is found, the gaze attends to it and explores it with a sequence of saccades and foveal fixations. In humans, single visual fixations can take 100–600 ms [29], thus indicating rather big differences in the rate at which input of sensory information is updated in the sequential visual process.

Bats construct their auditory world by also utilizing a sequence of beam movements and fixations similar to the “fixate and saccade“ strategy in vision [22]. Sequential sampling of two edges of a gap has been already demonstrated by Surlykke et al. (2009), who trained *E. fuscus* to fly through an opening in a fine net and measured the apparent beam direction in the horizontal plane. The information contained in each pulse-echo train can be compared to the information gained by each foveal fixation in the visual process. One difference between these systems lies in the speed at which updates can occur; the updating rate for succeeding acoustic fixations in bats is much faster than in the human visual system. When searching for prey in open space, and when navigating along edges, bats produced signals with intervals around 90–100 ms, corresponding to approximately 10 updates per second. During the approach of the array pulse intervals were even shorter, and similar to an approach towards a net opening, a landing site, or prey [10], [14], [15]. In humans the updating rate is determined by the duration of the fixation time (100–600 ms) and reaches values from ∼ 2–10 updates per second [29].

Good visual search performance is essential for survival, hence many efficient strategies for selecting fixation points have evolved in mammals [30]. The scanning angles covered by head and eye movements in the human visual field are task-dependent [21]. Depending on whether humans are looking at a scene or searching a target within that scene, their scanning behavior can greatly differ [21], [31]. In bats, the sequential sampling of the environment by “acoustic gaze saccades” or scanning movements of the sonar beam also varied according to the task being performed.

Bats searching for prey in open space often made alternating acoustic gaze saccades by head movements. Sound sequences with irregular or no scanning movements were also recorded. The type, spatial distribution, and abundance of prey may determine which scanning behavior produces the highest success rate in a given situation (Figure 6).

In the three edge situations, scanning behaviors were found that shared similarities to those recorded in open space. Often, the beam was directed back and forth but also irregular scanning or no scanning was observed (Figure 6). In this situation, the bats had to perform two tasks in parallel: spatial orientation and prey detection. Site-dependent differences in scanning angle indicate that the environment had an influence on the scanning behavior of the bats, thus documenting task-specific behavior. At the farm, bats kept acoustic contact with the house wall on their left side by primarily moving their beam up and down. In the garden the bats circled while foraging, and made mainly right/left scanning movements. Before flying a curve to the right the bats occasionally aimed their sonar beam in this intended direction (Figure 4A–E). This “foresight” behavior in flight direction has already been described [11]. When flying along the forest road, bats utilized diagonal scanning movements, which may indicate the use of vegetation edges on both sides of the road for spatial orientation. The scanning movements also increased the search volume, which may improve the chance for prey detection.

Bats switch to approach behavior in three different echolocation situations: obstacle avoidance, prey pursuit, and landing. Here we could only investigate the obstacle avoidance behavior of bats approaching the microphone array. During the approach to the array, the scanning angles were reduced and the beam maxima of succeeding pulses stayed on the target of interest for a longer time, e.g., one pipistrelle bat flew through the microphone array and moved the beam with small changes of the scanning angle around the sector which was later passed. (Figure 3A, center plot). For the two other approach situations, landing and prey pursuit, previous studies suggest that bats lock their beam onto the target they are interested in [10], [15]. This “locking in” on a target with the sonar beam can be compared to smooth pursuit movements in vision which keeps the target of interest on the fovea, and compensates for angular displacements produced by movements of either the target, the observer, or both [32].

We found that echolocating pipistrelle bats use a “saccade and fixate” strategy similar to vision. Since alternating signal amplitudes are also found in other bat species it is most likely that this finding can be generalized for all bats. By using these scanning movements bats are capable of finding and exploring targets in a wide search cone pointing in flight direction. We also found that the scanning behavior is task specific. However, more data collected with larger arrays are needed to fully understand how the scanning behavior is connected to specific echolocation tasks.

## Materials and Methods

### Ethics Statement

No specific permits were required for the described field studies since only sound recordings were made and no specimen were sampled and/or handled. No specific permits were required for the locations where recordings took place. Private land was accessed with the permit of Laurent Arthur from the Muséum d’Histoire Naturelle de Bourges, France. Field studies did not disturb endangered or protected species.

### Animals and Recording Sites

**Figure 7:**
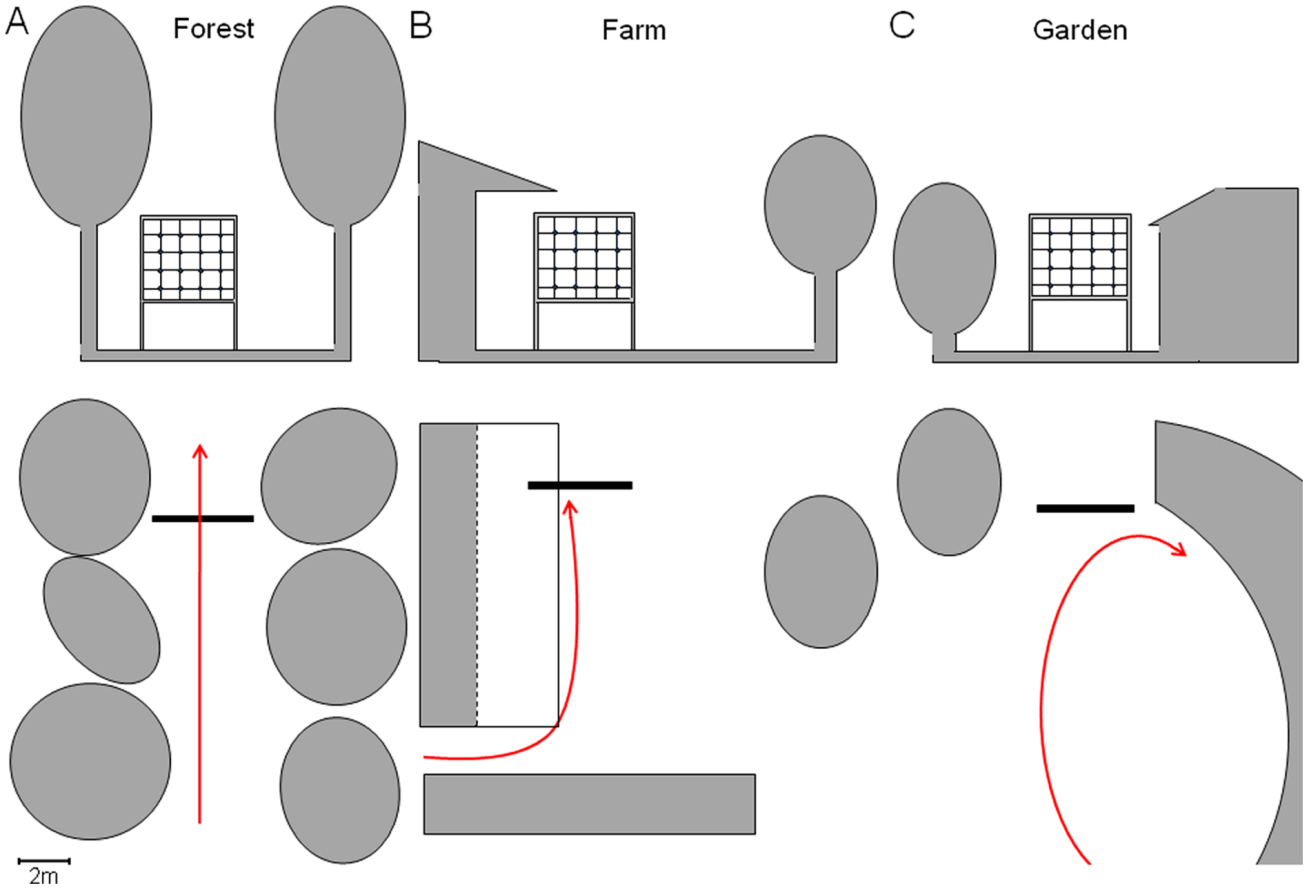
Recording sites in edge space. Side (upper row) and overhead views (lower row) of the three recording sites representing (A) forest road, which was lined with high deciduous trees, (B) farm, where the bats flew towards the microphone array along a house wall on their left side, (C) garden, where the bats circled in front of the array with a concave house front on their right side. Red arrows indicate typical flight paths.

Edge space recordings of the common pipistrelle bat (*P. pipistrellus*) were made at three locations in Central France (referred to as forest, farm, and garden) between June 30–July 27, 2009, and October 5–October 9, 2010, between 21∶00 and 02∶00 hours (MEZ). The three recording sites were chosen to represent three different echolocation scenes: bats flying along a house wall on their left side (farm; Figure 7B), bats circling in a garden in front of a barn (garden; Figure 7C), and bats flying along a straight forest road with dense vegetation on both sides (forest; Figure 7A). At each recording site and recording date several individuals with different terminal frequencies were observed, however the possibility of pseudoreplication of individuals to some degree cannot be completely excluded. At all three sites the microphone array was positioned perpendicular to the flight paths and only bats flying towards the array were recorded. On the farm, the lowest microphone row was positioned 1.6 m above a stony ground surface, the house wall on the left side was ∼ 2 m away, with the house roof partially covering the array at a height of 6.5 m. A few trees on the right side were more than 10 m away at this site. In the garden, the lowest microphone row was 1.7 m above pasture-covered ground with ∼ 1 m distance to the corner of a barn on the right side and 1–2 m to the bushes on the left. The bats circled in the more open area in front of the barn and the array. At this location, bats never flew past the array. In the forest, the array was positioned on a tarmac forest road forming a 10 m wide gap. The distance of the array to the forest edge at the left was ∼ 2 m and ∼ 4 m to the right. The lowest microphone row was 1.6 m above the road with foliage-covered ground on both sides.

Open space recordings of *P. pipistrellus* producing typical long open space signals were obtained in a grassland habitat next to a lake shore near Tübingen, Germany, on June 2, 2007, from 23∶00 to 23∶30 hours (MEZ).

### Experimental Setup

Recordings in the three different edge space situations were made using a vertical planar microphone array with 16 microphones arranged on nylon strings (ø 0.7 mm), facing perpendicular to the array plane, and forming a 4×4 grid. The strings were attached to an aluminum frame (4×4 m). The microphones were equally spaced 0.8 m apart on both the horizontal and vertical axis (Figure 1). We used nearly omnidirectional Knowles (FG-23329, Itasca, IL, USA) microphones with known angular sensitivity at different frequencies fixed in small custom-made housings. The recorded signals were amplified using a custom-made amplifier. After starting the recording manually, the signals of each of the 16 microphones were digitized by two 8-channel National Instruments (NI-PXI 6123) cards at 500 kHz sampling rate and fed into a ring buffer using custom-made software (in 2009 SIMI-MOTION version 7.5.0.288 and in 2010 LabView, National Instruments Corporation, Austin, TX, USA). After stopping the recording, the four last seconds in the buffer were stored on a laptop as Waveform Audio (.wav) files. For the single microphone recordings in open space, we used a custom-made ultrasonic microphone (PC-Tape microphone, Animal Physiology, University of Tübingen, Germany) with a flat frequency response of ±5 dB from 20–130 kHz. The data were digitized at 16-bit and stored on a laptop using a sampling rate of 480 kHz. Array recordings in open space were not reasonable, as the chances of recording a bat in search flight approaching the array directly would be too low.

### Database

From edge space recordings made using the microphone array, 32 flight sequences of pipistrelle bats containing 402 calls were analyzed. Sequences were chosen based on good signal quality (good signal-to-noise ratio at all 16 receivers) and a favorable flight path towards the microphone array. In front of the array the bats switched from search to approach behavior. We defined the beginning of the approach when two out of three sound parameters (sound pressure level, duration, pulse interval) exhibited continuous decline. 159 of the 725 calls were classified as approach calls. Only signals that were emitted at least 3 m away from the array were classified as search calls (476 signals). With this criterion we assured that no approach signals were mistaken for search signals. For the open space recordings, 22 flight sequences with 225 calls were chosen for analysis.

### Flight Path Reconstruction

Flight paths in front of the array were reconstructed using a custom-made Matlab (Mathworks, Natick, MA, USA) script to calculate the position of the bat at signal emission by using the time of arrival differences (TOADs) between microphones. The TOADs between the upper left array microphone and each of the other 15 microphones were computed by cross correlating the same echolocation call. The position of the sound source was then computed using a least-squares approximation [33]. In a test with a stationary ultrasonic speaker emitting a bat-like 10 ms long FM sweep from 80–10 kHz at different positions in front of the array we found that the positioning error in all three dimensions was no more than 2–3% of the distance to the array.

### Signal Analysis

The three signal parameters duration, pulse interval, and terminal frequency were measured in color spectrograms (FFT 512, Hann window, dynamic range of 90 dB) using custom-made software (Selena, University of Tübingen, Germany). Due to auto-padding and time interpolation, a resolution of *t* = 0.05 ms and *f* = 215 Hz was reached for both the array and single microphone recordings. The beginning and end of signals in spectrograms were defined using the criterion of −6 dB below best amplitude.

### Calculation of Sonar Beams and Aiming

The TOAD positions along with the corresponding time stamps for each signal were used as input by Sonarbeam [34], a Matlab-based software, to calculate a polar graph of the sonar beam from the bat’s perspective with color-coded SPLs (Figure 1A). Geometrical spreading loss, atmospheric attenuation, and the individual microphone angular sensitivity were each accounted for.

For each reconstructed beam, the direction of maximal intensity was computed and displayed as vector on the flight path with colors discerning between search calls (blue) and approach calls (red) (Figure 1B–E). The reconstructed beam maxima are referred to as real, when beam maximum values fell within the array, or as apparent, when the maximum values fell either at the border or outside of the array. Preliminary tests with an artificial sound source indicated that the accuracy of beam reconstruction was sufficient to determine changes in angular orientation of the sonar beam. Position errors for the beam maximum of up to 15° were measured [35]. Real and/or apparent beam maxima of succeeding pulses were used to calculate the scanning angles. The scan path which connects successive beam maxima indicates scanning movements (Figure 3). The angular direction of scan path sections between calls was determined and displayed in six 30° bins (Figure 4).

### SL Determination

For the calculations of search phase source levels (SL), only calls of bats flying towards the array and emitted between 3–10 m were used. The frequency range was limited to 40–60 kHz and sound pressure levels (SPL) are given in dB SPL *re* 20 µPa at 1 m (rms). Only 141 of the recorded search calls were centered within the array plane in such a way as to ensure that only real beam maxima, and not apparent beam maxima, were measured. Statistically, the apparent source levels (ASLs) did not differ from the SLs (e.g. for the forest site: F_1,196_ = 0.44, *p*>0.5), therefore both ASLs and SLs were included in analyses.

### Statistics

Statistical analysis was performed in JMP (SAS Institute Inc., Cary, NC, USA). To test for differences of signal parameters at the three recording sites, a one-factorial ANOVA was performed followed by a post-hoc Tukey-Kramer test using standard significance criteria (*p*≤0.05). To avoid pseudoreplication when calculating the mean signal parameters from sequences containing a different number of signals, only the mean of each sequence was determined and used for further analyses.
